# Characterization of fugitive emissions of SLS 3D printing

**DOI:** 10.1080/15459624.2026.2722276

**Published:** 2026-09-25

**Authors:** Connor Krause, Judy Su, Doug Daniels, Yi-Hsuan Chen, Candace Su-Jung Tsai

**Affiliations:** a Department of Environmental Health Sciences, Fielding School of Public Health, University of California, Los Angeles, Los Angeles, California; b California NanoSystems Institute, University of California Los Angeles, Los Angeles, California; c Data Science Center, University of California Los Angeles Library, Los Angeles, California

**Keywords:** Exposure monitoring, in vitro cell study, industrial hygiene, nanoparticles

## Abstract

This study investigated airborne emissions and potential health risks associated with selective laser sintering (SLS) 3D printing using Nylon-12 powder. Particle size characterization, *in vitro* cell studies, and analytical techniques were employed to examine particulate exposure during SLS 3D printing processes. Pure Nylon-12 powder, hardened nylon, sintered material, and emitted particles during 3D printing were characterized using microscopy, spectroscopy, and other techniques. Multiple methods were utilized to collect emitted particles for further analysis. Airborne particle concentrations associated with 3D printing and the work area were monitored in real-time using particle spectrometers, which measured particle number concentrations ranging from 10 nm to 10 *μ*m in mobility sizes. During the *in vitro* studies, THP-1 human monocytic leukemia cells were exposed to four concentrations of Nylon-12 powder suspension (10, 100, 500, and 1,000 *μ*g/mL) assessing cytotoxicity and inflammatory response through 3-(4,5-dimethylthiazol-2-yl)-5-(3-carboxymethoxyphenyl)-2-(4-sulfophenyl)-2H-tetrazolium (MTS) assay and Tumor Necrosis Factor Alpha (TNF-Αlpha) enzyme-linked immunosorbent assay (ELISA). Cell toxicity investigations revealed non-linear viability reduction patterns, with significant decreases at higher concentrations (1000 *μ*g/mL, *p* = 0.0207), but not significant at the intermediate doses (100 and 500 *μ*g/mL). The TNF-α assay results indicated a dose-dependent inflammatory response to Nylon-12 powder exposure. The highest TNF-α concentration (0.088) was observed in cells exposed to 1000 *μ*g/mL, while the lowest concentration (0.085) was found in the 10 *μ*g/mL treatment group. Elemental composition analysis identified silicon, aluminum, and copper as consistent elements in pure Nylon-12, sintered material, hardened nylon, and emitted particles collected during sampling, indicating the presence of nylon particles in the airborne emission. Air sampling showed variable particle dispersion during printing phases, with respirable and nanoparticles ranging from 48.97 to 194.9 μg/m^3^. NanoScan SMPS data indicated elevated nanoparticle concentrations, peaking at 6,848 particles/cm^3^ during printing. These findings underscore the importance of monitoring and controlling airborne emissions in 3D printing environments to mitigate potential biological toxicity and develop safer practices for this technology.

## Introduction

3D printing is a subset of additive manufacturing that has emerged as a versatile manufacturing technique in both industrial and personal applications. This process encompasses various methods, including stereolithography (SLA), fused deposition modeling (FDM), and selective laser sintering (SLS), each with its unique advantages and potential risks (Gümperlein et al. 2018; Ngo et al. 2018).

FDM and SLS printing involve heating materials beyond their melting points, potentially releasing toxic substances, including volatile organic compounds (VOCs) and ultrafine particles, into the surrounding environment (Unwin et al. 2013; Scungio et al. 2017). These emissions have been identified as potential health risks to workers and users, particularly in poorly ventilated spaces (Steinle 2016; Stefaniak et al. 2019).

Several studies have investigated the emission of ultrafine particles and VOCs from desktop printers; these investigations have reported varying concentrations of particles and VOCs depending on filament type, printer model, and sampling location (Stephens et al. 2013; Azimi et al. 2016; Deng et al. 2016; Floyd et al. 2017). One study observed ultrafine particle concentrations reaching up to 80,000 particles/cm^3^, with numerous VOCs detected, including respiratory sensitizers such as toluene, ethylbenzene, and formaldehyde (Kim et al. 2015). Additional research has further characterized these emissions, confirming the presence of potentially harmful substances including benzene and styrene (Gu et al. 2019; Wojtyła et al. 2020).

Limited research has been conducted on the subsequent biological responses to exposure to 3D printer emissions. A study found that exposing rats to ABS printing emissions via inhalation at a concentration average of 240 μg/m^3^ for 4 h a day across varying number of days (1, 4, 8, 15, and 30) would increase macrophage response in the rat with minimal increase in pulmonary toxicity (Farcas et al. 2019a). An *in vitro* study using human small airway epithelial cells revealed dose-dependent responses in cytotoxicity, oxidative stress, apoptosis, and necrosis with six varying exposure concentrations developed through dilution of captured airborne particulates during printing (Yi et al. 2016). These studies focused primarily on emissions from ABS filament used in FDM printing.

While some research has been conducted on FDM and SLA printers, there is a notable gap in the literature regarding emissions and potential exposure risks associated with SLS printers. SLS printers differ from FDM and SLA printers in their use of fine powder (commonly nylon) as the printing material and their more enclosed design (Goodridge et al. 2012). This gap in knowledge is particularly concerning given the increasing adoption of SLS technology for printing large-scale 3D items in various industries, from aerospace to medical device manufacturing (Olakanmi et al. 2015; Schmidt et al. 2017).

The growth of the 3D printing industry has underscored the need for focused studies on the potential health risks associated with these systems (Zontek et al. 2017). As technology becomes more accessible in both industrial and educational settings, understanding the nature and extent of emissions is crucial for developing appropriate protection measures and guidelines (Short et al. 2015).

The potential health effects of nanoparticle exposure from 3D printing processes have become a concern in occupational health (Warheit and Sayes 2015; Schulte et al. 2019). The small size of nanoparticles allows them to penetrate deep into the lungs and potentially enter the bloodstream, raising concerns about their systemic effects (Oberdorster et € al. 2005). Inhalation of these particles may lead to respiratory issues, cardiovascular problems, and potential long-term health effects (Bourell et al. 2020). Recent workplace measurements have highlighted the importance of monitoring ultrafine particle exposure in 3D printing environments (Scungio et al. 2017; Viitanen et al. 2017; Zontek et al. 2017).

This study aimed to address these knowledge gaps by investigating the emissions from a Formlabs Fuze 1 SLS printer using Nylon-12 powder. The objectives were to: 1) characterize the size distribution and morphology of airborne particles emitted during SLS printing, 2) assess the potential toxicity of Nylon-12 powder through *in vitro* cell studies, 3) analyze the chemical composition of emitted particles to confirm their origin from the printing process, and 4) evaluate the variability in particle dispersion during different printing and environmental conditions.

By employing a comprehensive approach combining air sampling, microscopy, chemical analysis, and *in vitro* toxicology, this study seeks to provide insights into SLS printer emissions. The findings from this research will not only enhance our understanding of potential exposure and biological toxicity associated with SLS 3D printing.

## Methodology

### Study design

Air sampling was conducted before, during, and after printing operations to capture the full range of emissions. Additionally, an *in vitro* study was conducted where THP-1 cells were exposed to various concentrations of Nylon-12 powder to assess potential biological effects.

### Printer operation, air sampling, and air monitoring

The Formlabs Fuze 1 SLS printer (Formlabs Fuze 1, Formlabs, Somerville, Massachusetts, USA), utilizing Nylon-12 powder as the printing material, was operated following a standardized protocol by the manufacturer. Each printing procedure began with design selection that was downloaded to the printer for preparation, followed by heating to 180 C for the preprint phase. The printing process typically lasted approximately 12 hr, followed by a cooldown phase necessary for safe handling of printed objects.

Air monitoring was conducted for 9 hr during printing. A total of six rounds of data collection were performed, with Prints 1 and 2 sampled during springtime evenings and Prints 3 to 6 sampled during summertime mornings.

To measure mass concentrations and perform particle characterizations, airborne particles were collected with a nanoparticle sampler, the Tsai Diffusion Sampler (TDS), and two National Institute for Occupational Safety and Health (NIOSH) Manual of Analytical Methods (NMAM), 0500 and 0600. The TDS, designed for nano and respirable particles, collects particles onto a 25 mm diameter polycarbonate (PC) filter with 0.22 μm pore size. A carbon-coated 400-mesh transmission electron microscopy (TEM) grid is attached at the center of the filter, allowing direct analysis of collected particles (Tsai and Theisen 2018). The TDS was connected via tubing to a GilAir Plus personal air sampling pump, set to a flow rate of 1 L/min, which is the upper range of the sampling flow rate. This configuration facilitates subsequent analysis of particle morphology and composition.

The NMAM 0500 and 0600 methods utilized 37 mm PVC filters in sampling cassettes with SKC pumps calibrated to 1.5 L/min and 2.5 L/min flow rates, respectively, for particle collection. The NMAM 0600 method used an aluminum cyclone to separate respirable particles for collection. Pre and post calibrations of all air pumps, including those used for the TDS and NMAM methods, were conducted using a MesaLabs DryCal Defender 510. All sampling filters were conditioned, and pre and post weights were measured using a microbalance later to calculate total mass collected on the filter and mass concentrations.

Real-time monitoring of airborne emissions was conducted using two direct reading devices: a NanoScan scanning mobility particle sizer (SMPS) (Model 3910, TSI Inc., Shoreview, Minnesota, USA) which measures particle sizes from 10 to 420 nm, and an optical particle sizer (OPS) (Model 3330, TSI Inc., Shoreview, Minnesota, USA) which measures particles from 0.3 to 10 *μ*m. These devices provided particle size and concentration measurements every minute throughout the preprint and printing process.

All samplers were positioned in proximity to the SLS printer without interfering with normal operations. Figure 1A shows the top view location of the printer within the room with sampling locations, while Figure 1B shows the side view of sampling and monitoring equipment in relation to the printer itself, with sampling equipment placed on a cart in order to access desired sampling locations. The workflow of this study is presented in Figure 1C. Temperature and humidity were monitored using the OPS and supplemented with local weather data from Weather Underground as shown in Figure 5.

### Particle characterization

Particle characterization involves multiple analytical techniques. A Supra 40VP scanning electron microscope (SEM) (Zeiss, Oberkochen, Germany) and a Tecnai T12 transmission electron microscope (FEI, Hillsboro, Oregon, USA) were used to analyze pure Nylon-12 powder, sintered material (loose nylon powder collected off of hardened nylon), hardened nylon (the final printed nylon product), and airborne particles collected on the sampling media (filter and TEM grid) of TDS. SEM samples were sputter-coated with a thin layer of gold (4nm) to reduce charging effects and improve contrast during imaging and imaged at 20kV with a SE2 detector. TEM grids were removed from the collection PC filter with tweezers and imaged at 120kV. These visualization methods allowed for the determination of physical sizes, compositions, and morphology of collected particles. Energy-dispersive X-ray spectroscopy (EDX) analysis was performed on pure Nylon-12 samples and airborne particles to determine their elemental composition and confirm the origin of emitted particles. Dynamic light scattering (DLS) was used to measure the hydrodynamic diameter of Nylon-12 particles in the solution used for cell exposure studies.

### In vitro *cell study*

THP-1 human monocytic leukemia cells from ATCC (Manassas, Virginia, USA) were cultured in RPMI-1640 medium supplemented with fetal bovine serum, penicillin-streptomycin, and beta-mercaptoethanol. Cells were exposed to Nylon-12 powder concentrations ranging from 10 to 1,000 *μ*g/mL for 24 hr. Before cell exposure, the Nylon-12 powder was tested for endotoxin contamination using the Thermo Scientific Pierce Chromogenic Endotoxin Quant Kit.

Cell toxicity was evaluated using two unique assays. The 3-(4,5-dimethylthiazol-2-yl)-5-(3-carboxymethoxyphenyl)-2-(4-sulfophenyl)-2H-tetrazolium (MTS) assay assessed cell viability by measuring formazan absorbance at 490nm using a SpectraMax M5 microplate reader. Cytokine response was assessed by measuring tumor necrosis factor alpha (TNF-α) levels in the cell culture media supernatant using ELISA following the manufacturer’s guidelines.

A one-way analysis of variance (ANOVA) was used to identify statistical significance between each dose used in the cell study. A t-test was also applied to compare across doses to determine significance.

## Results

### Material characterization

Following SEM and TEM imaging, approximate particle sizes were identified to range from 10 nm to 30 μm across pure Nylon-12 powder, sintered material, hardened nylon, and airborne samples, as seen in Figure 2. Pure Nylon-12 powder samples (Figure 2A and B) demonstrated both individual particles and agglomerates, with smaller particles often adhering to the surface of larger ones. Hardened nylon products retrieved from the 3D-printed object (Figure 2C) exhibited similar morphology to pure powder, with loose particles visible on the surface and around the hardened nylon printed material.

Airborne samples collected by TDS during printing (Figure 2D–F) showed comparable size distributions and agglomeration patterns to pure powder samples as seen in Figure 2A and B. Figure 2D highlighted the presence of nanoparticle agglomerates in the atmosphere around the printer during operation, with individual sizes as small as 10nm observed in the agglomerate. Figure 2E displayed particles of varying sizes, including larger particles up to 5 μm, while Figure 2F showed agglomerations of particles around 1 μm in size.

The DLS analysis of the Nylon-12 powder suspension used for cell exposure studies revealed a peak hydrodynamic diameter of 257.95 nm, indicating potential agglomeration in the liquid suspension.

### In vitro *cell study*

The MTS assay results demonstrated varying effects of Nylon-12 powder exposure on THP-1 cell viability after 24h (Figure 3). Statistical analysis using one-way ANOVA revealed significant differences among the treatment groups (*p*= 0.00068). The highest dose (1,000 *μ*g/mL) resulted in a significant reduction in cell viability, with a survival rate of 75.98% compared to the control. Conversely, the 10 *μ*g/mL dose resulted in an elevated level of cell viability (131.77% relative to the control).

The analysis of the data indicated that the control samples and the 10 *μ*g/mL dose group did not differ significantly from each other. The intermediate doses (100 and 500 *μ*g/mL) showed reduced cell viability compared to the control, but these differences were not statistically significant. The 1,000 *μ*g/mL dose group exhibited a statistically significant reduction in cell viability compared to all other groups, suggesting a concentration-dependent effect on cell viability. Pairwise comparisons revealed significant differences between 10 and 100 *μ*g/mL (*p* = 0.004), 10 and 500 *μ*g/mL (*p* = 0.00399), and 10 and 1,000 *μ*g/mL (*p* = 0.0207) doses.

The TNF-α assay results indicated a dose-dependent inflammatory response to Nylon-12 powder exposure as well (Figure 3). One-way ANOVA revealed highly significant differences among the treatment groups (*p* = 9.8 × 10^−8^). The highest TNF-α concentration (0.088) was observed in cells exposed to 1,000 *μ*g/mL, while the lowest concentration (0.085) was found in the 10 *μ*g/mL treatment group.

Further statistical analysis showed that the two lower doses (10 and 100 *μ*g/mL) did not differ significantly from each other in terms of TNF-α production (*p* = 0.185). Similarly, the two higher doses (500 and 1,000 *μ*g/mL) did not show significant differences between them (*p* = 0.500). However, there were statistically significant differences between the lower and higher dose groups, specifically between 10 and 500 *μ*g/mL (*p* = 0.00292), 10 and 1,000 *μ*g/mL (*p* = 0.0143), 100 and 500 *μ*g/mL (*p* = 0.00402), and 100 and 1,000 *μ*g/mL (*p* = 0.0151), indicating a clear increase in inflammatory response on treated cells at higher concentrations of Nylon-12 powder suspension.

### Elemental composition analysis

Using EDX analysis, silicon, aluminum, and copper were consistently identified in both pure Nylon-12 and airborne particles from printing, as seen in Figure 4. In pure Nylon-12 samples, silicon constituted between 1.5% and 9% across all three variations. Aluminum was found exclusively in the hardened nylon (2.89%) and pure powder (0.44%) samples, while copper was detected only in the sintered material (4.60%). In the print samples, silicon was present at 4.30% and 3.85% in Print Samples 1 and 2, respectively. Copper was also detected in both samples at similar levels (4.02% and 4.04%), and aluminum was found at 1.81% and 1.16%. Importantly, these elements were absent or present at much lower levels in background samples (Figure 4), confirming their association with the printing process. Carbon and oxygen percentages were removed due to their prevalence as primary elements across all samples. SEM samples were sputter-coated with a thin layer of gold to reduce charging effects during imaging; thus, the presence of gold is not attributed to the particles.

### Airborne particle number concentration analysis

As shown in Table 1, mass concentration measurements of particles collected on filters using the TDS yielded quantifiable increases across all six print samples, ranging from 48.97 to 194.9 μg/m^3^. By contrast, the mass concentrations of particles collected using the NMAM 0500 and NMAM 0600 methods showed quantifiable increases in mass concentrations less frequently (Table 1). With the NMAM 0500 method, the measured mass concentration increases were observed in only three out of six samples (Prints 2, 3, and 4), while the mass concentration of NMAM 0600 method detected an increase in only one sample (Print 4).

The mass concentrations of particles collected using the TDS method consistently yielded the highest mass concentrations among the three methods. The maximum TDS mass concentration was measured during Print 3 at 194.9 μg/m^3^ (SD: 30.79), while the minimum was recorded during Print 5 at 48.97 μg/m^3^ (SD: 12.12). The variability in TDS measurements across prints was considerable, with standard deviations ranging from 11.85 to 42.42 μg/m^3^.

For the NMAM 0500 method, quantifiable increases were observed in mass concentrations of particles collected during Prints 2, 3, and 4, with concentrations of 22.97 μg/m^3^ (SD: 21.06), 27.47 μg/m^3^ (SD: 13.74), and 55.50 μg/m^3^ (SD: 16.02), respectively. The NMAM 0600 method detected a quantifiable increase only in Print 4, measuring 33.37 μg/m^3^ (SD: 4.82).

Regarding particle number concentrations, the NanoScan SMPS measurements showed substantial variability across prints, ranging from 321.2 particles/cm^3^ (SD: 106.8) in Print 1 to 6,848 particles/cm^3^ (STD: 3,479) in Print 4. The OPS measurements, which capture larger particles, displayed less variation, with concentrations ranging from 6.81 particles/cm^3^ (SD: 1.710) in Print 2 to 48.03 particles/cm^3^ (SD: 17.36) in Print 4.

The NanoScan SMPS and OPS measurements indicated a two-phase pattern in total particle number concentrations during printing (Figure 5). For Prints 1 and 2, the data exhibited minimal variance in total concentrations initially, followed by an increase in variance and particle counts after roughly 200 min of sampling time. Prints 3 to 6 demonstrated a similar two-phase trend, although with varying peak concentrations and durations. In these later prints, both NanoScan SMPS and OPS total particle number concentrations indicated increasing total particle counts in phase 1, followed by a decrease in phase 2 as seen in Figure 5A.

The peak concentrations in particle concentrations from the NanoScan SMPS were typically observed just prior to the end of printing phase 1, while OPS peaks occurred during printing phase 2, suggesting a slight lag in larger particle emissions and formation of agglomerates.

Temperature measurements indicate an initial increase followed by relative stability across all prints, as presented in Figure 5B. During Print 5, the highest average temperature was recorded at 36 C, while during Print 2, the lowest average temperature was observed at 29 C. Outdoor temperatures and relative humidity showed inverse trends, with Prints 3 and 4 experiencing the highest temperatures and lowest relative humidity.

### Particle size distributions

The number concentration data collected by NanoScan SMPS data for Prints 1 and 2 showed that concentrations during printing were lower than the concentrations before printing started across all size fractions (10 to 420 nm) throughout the three phases, with overlapping standard deviations as seen in Figure 6A. In contrast, higher particle number concentrations were observed during Printing Phase 1 for particle sizes between 15.4 and 86.6 nm compared to background and Printing Phase 2 during Prints 3 to 6 (Figure 6B).

The number concentration data from the OPS showed similar trends, with higher concentrations at smaller cut points (0.35 to 0.475 μm) of particle size during printing phases compared to background, particularly for Prints 3 to 6. The most notable difference was observed at the 0.35 μm cut point, with up to 60 particles/cm^3^ difference between printing phase 1 and background in Prints 3 to 6 (Figure 6C and D).

## Discussion

This study provides valuable insights into the emissions and potential toxicity using an *in vitro* model associated with SLS 3D printing using Nylon-12 powder. These findings highlight the complex nature of particle emissions during the printing process and their potential biological effects.

### Nylon-12 particle characteristics and emissions

The consistent particle size range (10 nm to 30 μm) observed across pure Nylon-12 powder, hardened nylon, sintered material, and airborne samples suggests that the particles emitted during printing are indeed Nylon-12. The presence of nanoparticles as small as 10 nm in agglomerates is particularly concerning, as agglomerates containing particles of this size can potentially become individual particles when humans inhale and could penetrate deep into the respiratory system and even enter the bloodstream (Davis et al. 2019). Given nanoparticles found in Figure 6B were 50 nm and smaller, exposure should be avoided, since nanoparticles around 50 nm have been found to cause cell necrosis when the particles are able to deposit in the lung tissue and enter the cells (Sukhanova et al. 2018)

The DLS analysis revealed agglomeration of Nylon-12 particles in liquid suspension. The identified range was still an effective range for interaction to occur between cells and the nylon powder (Farcas et al. 2019a).

### Biological effects of Nylon-12 exposure in cells

The increased viability at lower doses (10 μg/mL) could indicate a potential hormetic response, where low levels of stress induce adaptive mechanisms in cells (Farcas et al. 2019b). However, the significant decrease in viability at the highest dose (1,000 μg/mL, *p* = 0.0207 compared to 10 μg/mL) clearly demonstrates the potential cytotoxicity of Nylon-12 particles at elevated concentrations.

The dose-dependent increase in TNF-α production corroborates the cytotoxicity findings and shows that Nylon-12 exposure can trigger inflammatory responses in cells. This inflammatory potential is particularly evident in the statistically significant differences between lower and higher dose groups (e.g., *p* = 0.00292 between 10 and 500 μg/mL, *p* = 0.0143 between 10 and 1,000 *μ*g/mL). Interestingly, while there was a clear step-up in inflammatory response from lower to higher doses, the lack of significant difference between the two highest doses (500 and 1,000 μg/mL, *p* = 0.500) suggests a possible plateau effect in the inflammatory response at higher concentrations.

These findings underscore the importance of considering both low-dose and high-dose effects when assessing the potential health impacts of Nylon-12 particles. The results have implications for respiratory health in occupational settings where chronic exposure to 3D printer emissions occurs (Stefaniak et al. 2019), highlighting the need for appropriate protection measures even at concentrations that may not immediately appear cytotoxic.

### Particle emissions and environmental factors

The two-phase pattern (Figure 5) observed in particle concentrations during printing suggests that emission characteristics vary throughout the printing process. The initial increase in particle concentrations could be attributed to the heating and melting of Nylon-12 powder, while the subsequent decrease might result from particle settling or changes in printer operation (Zontek et al. 2017).

The influence of environmental factors, such as temperature and humidity, on particle emissions highlights the complexity of managing exposure risks in real-world settings (Chýlek et al. 2021). The seasonal variations observed in this study underscore the need for comprehensive monitoring and control strategies that account for changing environmental conditions (Wojtyła et al. 2020). For example, in higher humidity environments, particles would be more likely to agglomerate, leading to a lower exposure to individual nanoparticles (He et al. 2019).

### Chemical composition of emitted particles

The EDX analysis confirms the presence of silicon (Si), aluminum (Al), and copper (Cu) in both pure Nylon-12 and airborne particles, providing strong evidence that the emitted particles originate from the printing process. The absence of these elements in background samples further supports this conclusion. However, the presence of these elements, which are not typically associated with pure nylon, raises questions about potential additives or contaminants in the Nylon-12 powder used for printing (Steinle 2016). Among these three elements found, the presence of Si raised concern since Si is a known hazard associated with many respiratory illnesses and toxic effects, including cancer when exposure occurs. When inhaled by humans, Si, a confirmed human carcinogen, damages lung tissue and increases the risk of lung cancer, silicosis, chronic obstructive pulmonary disease, and kidney disease (OSHA 2024). Additionally, exposing humans to a complex mixture of elements such as those found in this study can potentially cause more serious respiratory effects from the combination effects due to interactions between nanoparticles and other contaminants, such as those adsorbing an airborne pollutant and allowing it to be taken into cells of the human body (Forest 2021).

## Limitations

While this study provides valuable insights into the emissions and potential health risks associated with SLS 3D printing using Nylon-12 powder, several limitations should be acknowledged. The lack of environmental controls such as local exhaust ventilation in the lab, the operation of general ventilation systems, and open windows likely contributed to inconsistencies in particle concentrations and size distributions. Although this represents a real work environment, a more controlled setting would allow for better isolation of printer-specific emissions. Additionally, due to instrument limitations, air monitoring was restricted to 9 h, which may not capture the full extent of emissions during longer print jobs or cooldown phases.

The use of the THP-1 cell line in the *in vitro* studies, while providing useful insights, may not fully represent the complex interactions that occur in the human respiratory system. Future studies could benefit from using more advanced *in vitro* models or human subject studies. Furthermore, this study focused on a single SLS printer model, potentially limiting the generalizability of findings to different printer models or brands with varying emission profiles.

Addressing these limitations in future research will be crucial for developing a more comprehensive understanding of the potential health risks associated with SLS 3D printing and for establishing effective protection guidelines for this technology.

## Conclusion

This study provides quantitative evidence of nanoparticle and microparticle emissions during Selective Laser Sintering (SLS) 3D printing using Nylon-12 powder and offers insights into their potential biological effects. SEM and TEM analyses revealed a consistent particle size range of 10 nm to 30 μm across various Nylon-12 samples, including airborne particles. Real-time monitoring identified a two-phase pattern in particle emissions during printing, with NanoScan SMPS data showing elevated nanoparticle concentrations peaking at 6,848 particles/cm^3^. Mass concentration measurements using the Tsai Diffusion Sampler (TDS) yielded quantifiable increases across all six print samples, ranging from 48.97 to 194.9 μg/m^3^.

EDX analysis confirmed the presence of Si, Al, and Cu in both pure Nylon-12 and airborne particles, providing strong evidence that emitted particles originate from the printing process. *In vitro* studies using THP-1 cells demonstrated a non-linear dose-response in cell viability and a dose-dependent increase in TNF-Alpha production, indicating potential cytotoxicity and inflammatory responses to Nylon-12 particle exposure. Environmental factors, particularly temperature and humidity, were found to influence particle emissions.

The identification of unexpected elements in the Nylon-12 particles suggests the presence of additives or contaminants, which may contribute to the observed biological responses. These findings highlight the complex nature of emissions from SLS 3D printing and their potential health implications. The presence of nanoparticles in the emissions, combined with their demonstrated biological effects *in vitro* and the presence of several hazardous metal elements, underscores the importance of considering both particle size and composition when assessing potential health risks. A proper control measure, such as an enclosed ventilation system/booth equipped with a High-Efficiency Particulate Air (HEPA) filter, shall be designed and employed for this type of SLS printer to remove and capture airborne emissions, including particulates and volatile compounds during the printing process. An N-100 type of respirator for particulates shall be worn by operators as extra protection while performing tasks on or around the printer.

This research contributes valuable data to the growing body of evidence on 3D printer emissions, particularly in the understudied area of SLS technology. The results provide a scientific basis for understanding the potential occupational health risks associated with SLS 3D printing and highlight the need for further investigation into long-term exposure effects and the development of appropriate safety measures.

## Recommendations

From an industrial hygiene perspective, implementing real-time particle monitoring systems in 3D printing workspaces is recommended to identify periods of peak emissions and trigger appropriate ventilation responses. Future research should focus on conducting longitudinal studies in occupational settings where SLS printers are frequently used, including personal exposure monitoring and health surveillance of workers. Additionally, investigating particle emissions during different stages of the printing process, including material handling and post-processing, would provide a more comprehensive understanding of exposure risks. The Formlabs printer in this study is specifically designed to work in tandem with a Fuze Sift (Fuze Sift, Formlabs, Somerville, Massachusetts, USA) hood, of which there are no current research studies. A published study has demonstrated effective controls for airborne nanoparticles using certain types of fume hoods, such as biological safety cabinets or other similar hoods (Lo et al. 2015). The post-processing involved in this lab has employed a Fuze Sift high-efficiency hood (Formlabs, Somerville, Massachusetts, USA), showing successful containment of the nylon powder. Developing standardized testing protocols for 3D printer emissions to support the creation of evidence-based safety guidelines for additive manufacturing environments. Given the large quantity and powder materials used for 3D printing using SLS technology, control measures suggested in this study, enclosed ventilation equipped with a HEPA filter and a respirator, can be implemented for printers using other types of polymers. All 3D printers should explore the effectiveness of various emission control strategies, such as enhanced local exhaust ventilation systems or enclosed printing environments, to minimize worker exposure. Lastly, research on the potential for particle resuspension during cleaning and maintenance activities, as well as the synergistic effects of multiple 3D printers operating simultaneously in shared workspaces, would contribute valuable insights to improving occupational health and safety in 3D printing environments.

## Figures and Tables

**Figure 1.**
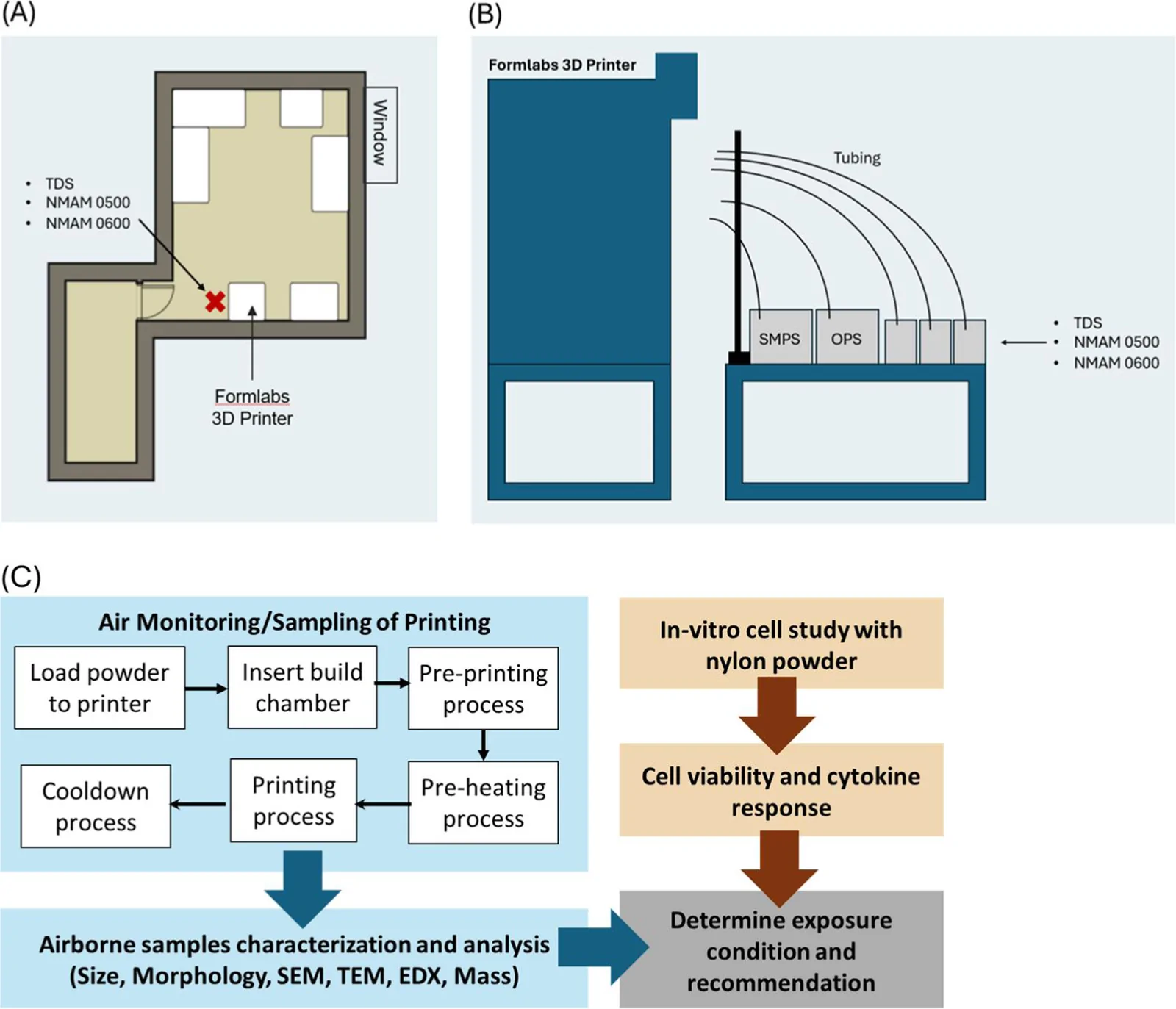
Schematic of the printing and particle collection locations. (A) Top view of room setup with printer location. (B) Side view of experimental monitoring setup schematic. (C) Study workflow chart.

**Figure 2.**
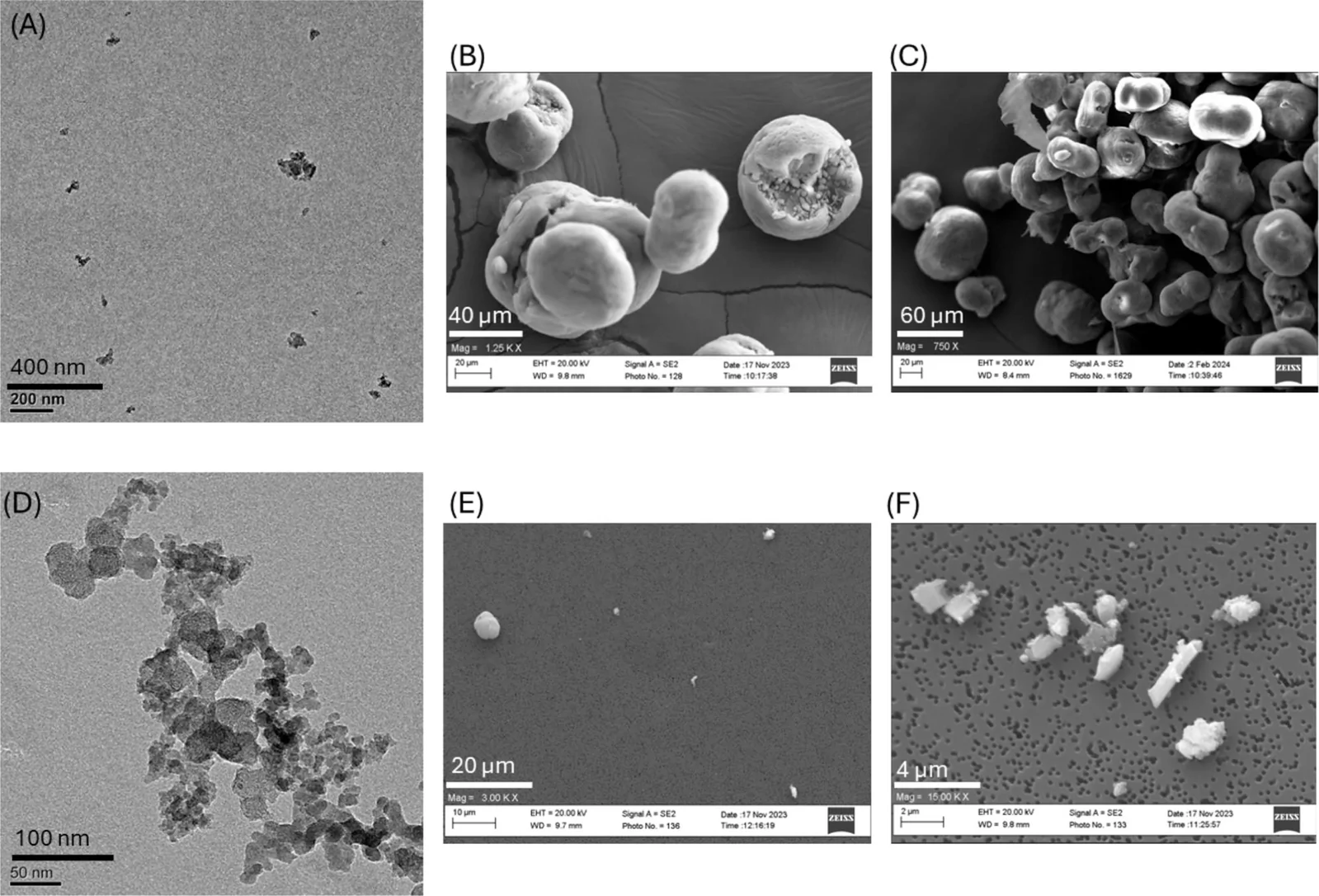
SEM and TEM imaging of pure Nylon-12 and airborne sampling during printing. (A) TEM image of pure Nylon-12; (B) and (C) SEM images of hardened products from Nylon-12. (D) TEM image of airborne particles captured during printing by TDS; (E) and (F) SEM images of airborne particles captured during printing by TDS.

**Figure 3.**
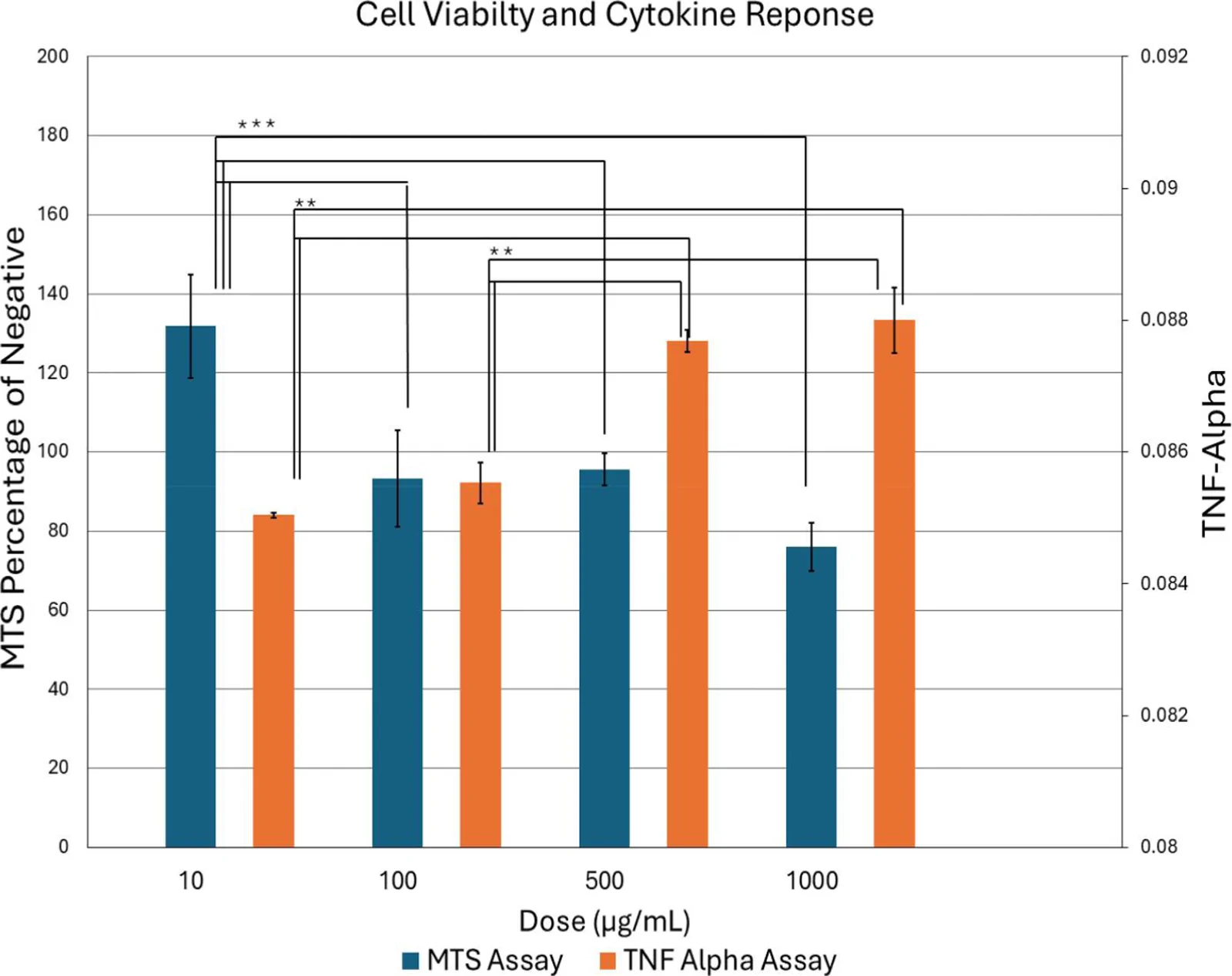
MTS Assay for cell viability and TNF-Αlpha Assay for Cytokine response results showing responses of cells to four different dose exposures to Nylon-12. over a joining bar indicates *p*< 0.05 between the two joined exposure doses. Multiple stars refer to the number of significant conjoining lines below.

**Figure 4.**
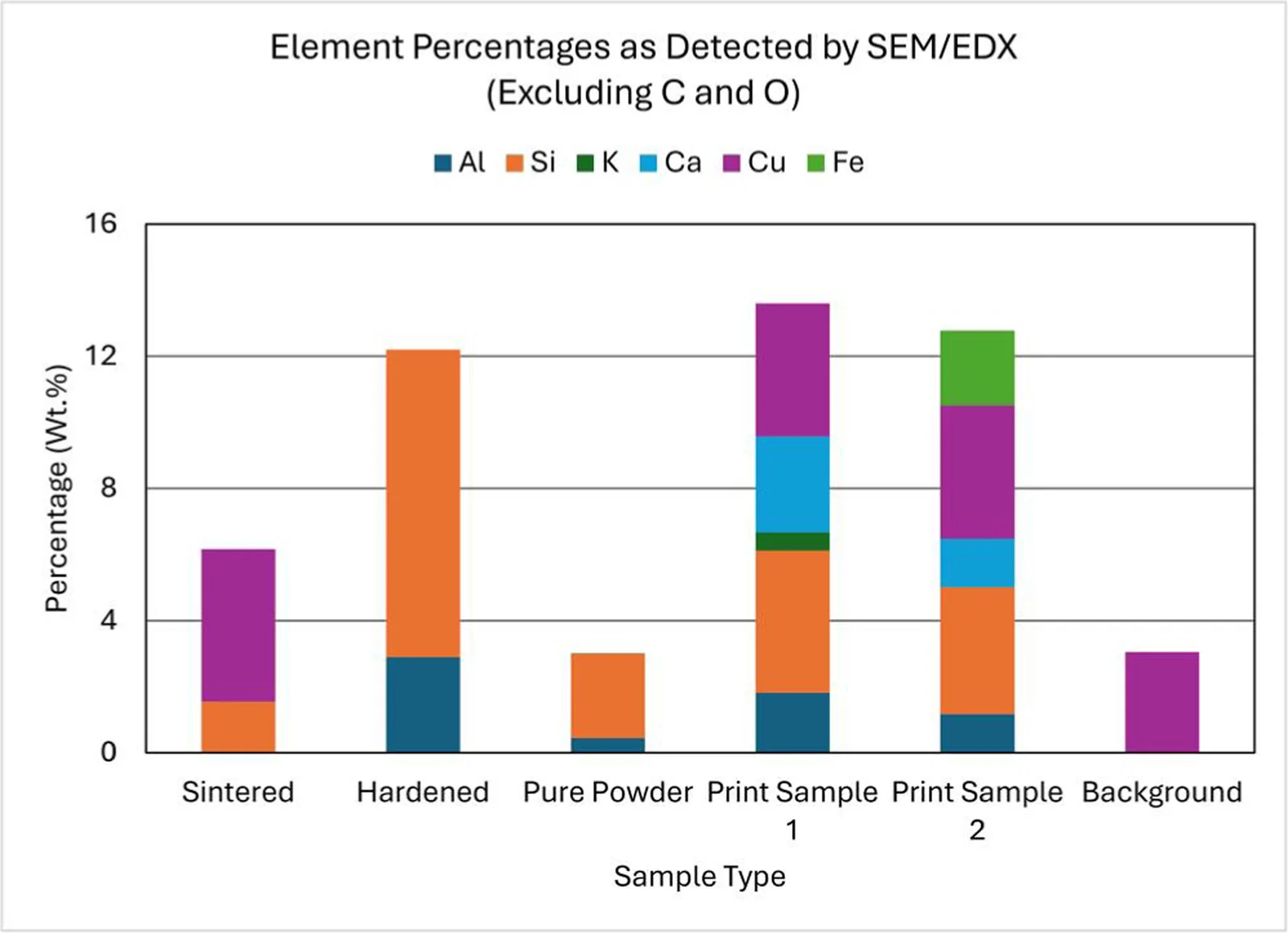
EDX chemical composition with adjusted percentages. Carbon, oxygen, and gold are excluded due to consistent presence in all prints, as gold is from sample sputter coating, and carbon and oxygen are the primary elements. Note: Wt.% is weight percentage.

**Figure 5.**
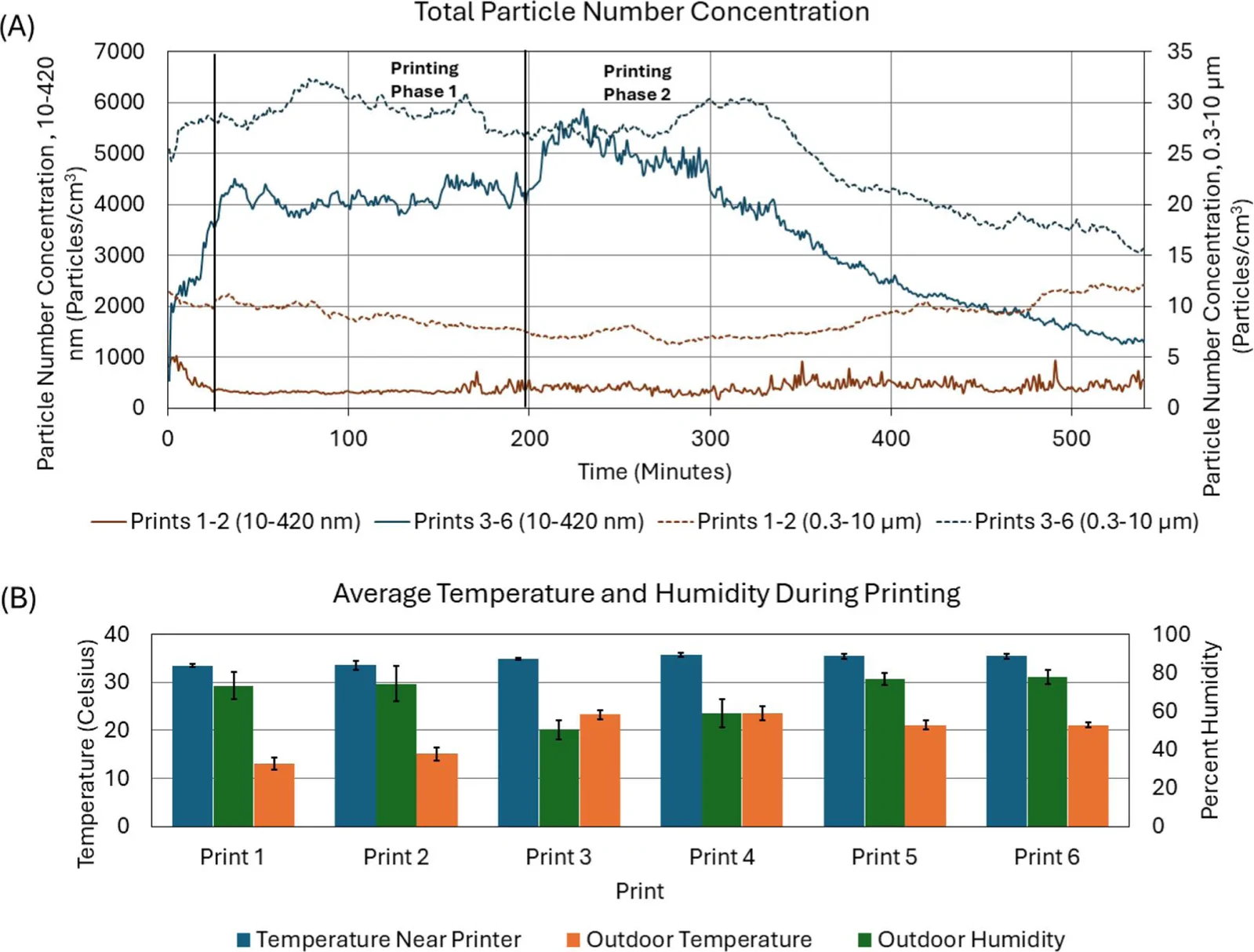
Total concentrations over time across all prints and environmental factors. (A) NanoScan SMPS and OPS particle concentrations over time, with bars representing the two unique phases of printing trends. (B) Temperature data near printer (from OPS) and outdoors, and relative humidity outdoors.

**Figure 6.**
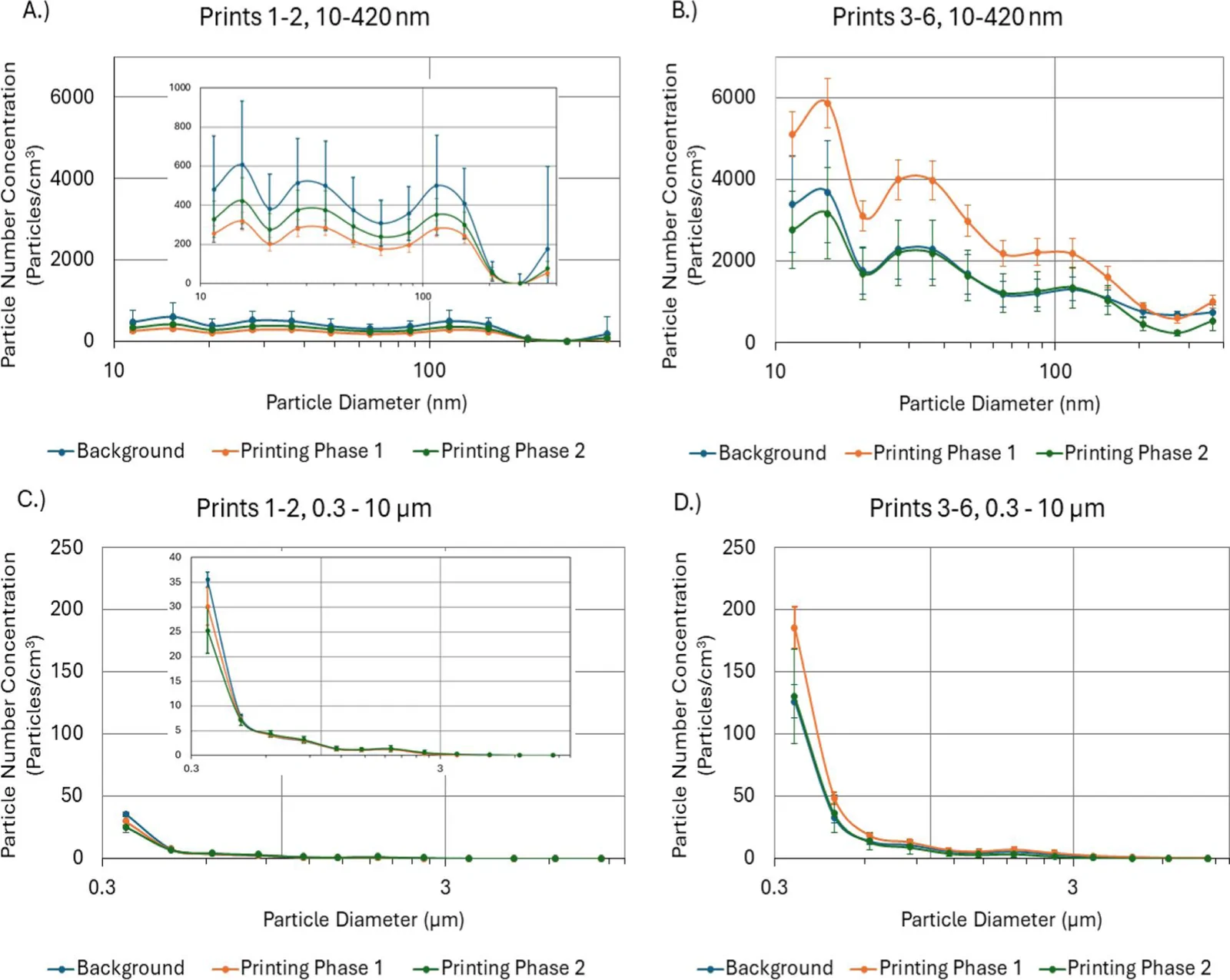
Particle size-fraction number concentrations from 10 nm to 10 μm monitored by NanScan SMPS and OPS for each prints. (A) Particle size fractions observed in Prints 1 to 2 from data collected using NanoScan SMPS, with additional scaling provided with inlayed graph. (B) Particle size fractions observed in Prints 3 to 5 from data collected using NanoScan SMPS. (C) Particle size fractions observed in Prints 1 to 2 from data collected using OPS, with additional scaling provided with inlayed graph. (D) Particle size fractions observed in Prints 3 to 6 from data collected using OPS.

**Table 1.** Mass concentrations and number concentrations for each print sampled with averages and standard deviations (STD).

Print	Mass Concentration (*μ*g/m^3^)						Number Concentration (particles/cm^3^)			
		
	TDS		NMAM 0500		NMAM 0600		NanoScan SMPS		OPS	
					
	AVG	SD	AVG	SD	AVG	SD	AVG	SD	AVG	SD

1	75.28	11.85	NI	NI	NI	NI	321.2	106.8	10.76	1.840
2	103.5	11.96	22.97	21.06	NI	NI	460.5	160.8	6.81	1.710
3	194.9	30.79	27.47	13.74	NI	NI	3701	677.2	26.19	9.268
4	76.23	20.79	55.50	16.02	33.37	4.82	6848	3479	48.03	17.36
5	48.97	12.12	NI	NI	NI	NI	1565	542.7	27.61	9.333
6	108.7	42.42	NI	NI	NI	NI	1996	1247	24.79	8.671

AVG-Average, SD-Standard Deviation, NI-No Increase.

## Data Availability

The data that support the findings of this study are available from the corresponding author, CT, upon reasonable request. All data (all original analyzed data and images) are stored in UCLA Box.

## References

[R1] Azimi P, Zhao D, Pouzet C, Crain NE, Stephens B. 2016. Emissions of ultrafine particles and volatile organic compounds from commercially available desktop three-dimensional printers with multiple filaments. Environ Sci Technol. 50(3):1260–1268. 10.1021/acs.est.5b04983 26741485

[R2] Bourell D, Beaman J, Wohlers T. 2020. History of additive manufacturing. Materials Park, OH: Digital Library, ASM International.

[R3] Chylek R, Kudela L, Pospısil J, Snajdarek L. 2021. Parameters influencing the emission of ultrafine particles during 3D printing. Int J Environ Res Public Health. 18(21):11670. 10.3390/ijerph182111670 PMC8582798 34770184

[R4] Davis AY, Zhang Q, Wong JPS, Weber RJ, Black MS. 2019. Characterization of volatile organic compound emissions from consumer level material extrusion 3D printers. Build Environ. 160:106209. 10.1016/j.buildenv.2019.106209

[R5] Deng Y, Cao S-J, Chen A, Guo Y. 2016. The impact of manufacturing parameters on submicron particle emissions from a desktop 3D printer in the perspective of emission reduction. Build Environ. 104:311–319. 10.1016/j.buildenv.2016.05.021

[R6] Farcas MT 2019a. Pulmonary and systemic toxicity in rats following inhalation exposure of 3-D printer emissions from acrylonitrile butadiene styrene (ABS) filament. Biostudies-literature, V1. https://www.ebi.ac.uk/biostudies/studies/S-EPMC7673646. Inhalation toxicology 20200901 11–12. 10.1080/08958378.2020.1834034 PMC7673646 33076715

[R7] Farcas MT 2019b. Acrylonitrile butadiene styrene (ABS) and polycarbonate (PC) filaments three-dimensional (3-D) printer emissions-induced cell toxicity. Toxicol Lett. 317:1–12. 10.1016/j.toxlet.2019.09.013 PMC7490755 31562913

[R8] Floyd EL, Wang J, Regens JL. 2017. Fume emissions from a low-cost 3-D printer with various filaments. J Occup Environ Hyg. 14(7):523–533. 10.1080/15459624.2017.1302587 28406364

[R9] Forest V 2021. Combined effects of nanoparticles and other environmental contaminants on human health—an issue often overlooked. NanoImpact. 23:100344. 10.1016/j.impact.2021.100344 35559845

[R10] Goodridge RD, Tuck CJ, Hague RJM. 2012. Laser sintering of polyamides and other polymers. Prog Mater Sci. 57(2): 229–267. 10.1016/j.pmatsci.2011.04.001

[R11] Gu J, Wensing M, Uhde E, Salthammer T. 2019. Characterization of particulate and gaseous pollutants emitted during operation of a desktop 3D printer. Environ Int. 123:476–485. 10.1016/j.envint.2018.12.014 30622073

[R12] Gümperlein I € 2018. Acute health effects of desktop 3D printing (fused deposition modeling) using acrylonitrile butadiene styrene and polylactic acid materials: an experimental exposure study in human volunteers. Indoor Air. 28(4):611–623. 10.1111/ina.12458 29500848

[R13] He Y 2019. Atmospheric humidity and particle charging state on agglomeration of aerosol particles. Atmos Environ. 197:141–149. 10.1016/j.atmosenv.2018.10.035

[R14] Kim Y 2015. Emissions of nanoparticles and gaseous material from 3D printer operation. Environ Sci Technol. 49(20):12044–12053. 10.1021/acs.est.5b02805 26402038

[R15] Lo L 2015. Performance of particulate containment at nanotechnology workplaces. J Nanopart Res. 17:435. 10.1007/s11051-015-3238-4 PMC4685715 26705393

[R16] Ngo TD, Kashani A, Imbalzano G, Nguyen KTQ, Hui D. 2018. Additive manufacturing (3D printing): a review of materials, methods, applications and challenges. Compos Part B Eng. 143:172–196. 10.1016/j.compositesb.2018.02.012

[R17] Oberdörster G, Oberdörster E, Oberdörster J 2005. Nanotoxicology: an emerging discipline evolving from studies of ultrafine particles. Environ Health Perspect. 113(7): 823–839. 10.1289/ehp.7339. Erratum in: Environ Health Perspect. 2010 Sep;118(9):A380. PMID: 16002369; PMCID: PMC1257642. PMC1257642 16002369

[R18] Olakanmi EO, Cochrane RF, Dalgarno KW. 2015. A review on selective laser sintering/melting (SLS/SLM) of aluminium alloy powders: processing, microstructure, and properties. Prog Mater Sci. 74:401–477. 10.1016/j.pmatsci.2015.03.002

[R19] OSHA. 2024. Silica, crystalline. https://www.osha.gov/silicacrystalline

[R20] Schmidt M 2017. Laser based additive manufacturing in industry and academia. CIRP Ann. 66(2):561–583. 10.1016/j.cirp.2017.05.011

[R21] Schulte PA, Leso V, Niang M, Iavicoli I. 2019. Current state of knowledge on the health effects of engineered nanomaterials in workers: a systematic review of human studies and epidemiological investigations. Scand J Work Environ Health. 45(3):217–238. 10.5271/sjweh.3800 PMC6494687 30653633

[R22] Scungio M, Vitanza T, Stabile L, Buonanno G, Morawska L. 2017. Characterization of particle emission from laser printers. Sci Total Environ. 586:623–630. 10.1016/j.scitotenv.2017.02.030 28196755

[R23] Short DB, Sirinterlikci A, Badger P, Artieri B. 2015. Environmental, health, and safety issues in rapid prototyping. Rapid Prototyping J. 21(1):105–110. 10.1108/RPJ-11-2012-0111

[R24] Stefaniak AB Jr. 2019. Evaluation of emissions and exposures at workplaces using desktop 3-dimensional printer. J Chem Health Saf. 26(2):19–30. 10.1016/j.jchas.2018.11.001 PMC6889885 31798757

[R25] Steinle P 2016. Characterization of emissions from a desktop 3D printer and indoor air measurements in office settings. J Occup Environ Hyg. 13(2):121–132. 10.1080/15459624.2015.1091957 26550911

[R26] Stephens B, Azimi P, El Orch Z, Ramos T. 2013. Ultrafine particle emissions from desktop 3D printers. Atmos Environ. 79:334–339. 10.1016/j.atmosenv.2013.06.050

[R27] Sukhanova A 2018. Dependence of nanoparticle toxicity on their physical and chemical properties. Nanoscale Res Lett. 13(1):44. 10.1186/s11671-018-2457-x. PMC5803171 29417375

[R28] Tsai CS-J, Theisen D. 2018. A sampler designed for nanoparticles and respirable particles with direct analysis feature. J Nanopart Res. 20(8):209. 10.1007/s11051-018-4307-2

[R29] Unwin J, Coldwell MR, Keen C, McAlinden JJ. 2013. Airborne emissions of carcinogens and respiratory sensitizers during thermal processing of plastics. Ann Occup Hyg. 57(3):399–406. 10.1093/annhyg/mes078 23091110

[R30] Viitanen A-K, Uuksulainen S, Koivisto AJ, Hämeri K, Kauppinen T. 2017. Workplace measurements of ultrafine particles—a literature review. Ann Work Expos Health. 61:749–758. 10.1093/annweh/wxx049 28810681

[R31] Warheit DB, Sayes CM. 2015. Chapter 1.2—routes of exposure to nanoparticles: hazard tests related to portal entries. In: Dolez PI, editor. Nanoengineering. Amsterdam: Elsevier.

[R32] Wojtyła S, Klama P, Śpiewak K, Baran T. 2020. 3D printer as a potential source of indoor air pollution. Int J Environ Sci Technol. 17(1):207–218. 10.1007/s13762-019-02444-x

[R33] Yi J 2016. Emission of particulate matter from a desktop three-dimensional (3D) printer. J Toxicol Environ Health A. 79(11):453–465. 10.1080/15287394.2016.1166467 PMC4917922 27196745

[R34] Zontek TL, Ogle BR, Jankovic JT, Hollenbeck SM. 2017. An exposure assessment of desktop 3D printing. J Chem Health Saf. 24(2):15–25. 10.1016/j.jchas.2016.05.008

